# Amyloidoma: A Case Report of Remote Insulin-Derived Amyloidosis in the Setting of Insulin-Dependent Diabetes

**DOI:** 10.7759/cureus.63525

**Published:** 2024-06-30

**Authors:** Daniel Duong, Tricia Westhoff-Pankratz, Amanda Frugoli, Samuel Pajuleras, Katie Ta, Brad Barrows

**Affiliations:** 1 Department of Graduate Medical Education and Family Medicine, Community Memorial Healthcare, Ventura, USA; 2 Department of Graduate Medical Education and Internal Medicine, Community Memorial Healthcare, Ventura, USA; 3 Department of Graduate Medical Education and Internal Medicine, Community Memorial Hospital, Ventura, USA; 4 Department of Graduate Medical Education, Community Memorial Hospital, Ventura, USA; 5 Department of Graduate Medical Education and Pathology, Community Memorial Healthcare, Ventura, USA

**Keywords:** amyloidoma, insulin injection, primary cutaneous amyloidosis, type i diabetes mellitus, congo red, remote insulin-derived amyloidosis

## Abstract

The incidence of insulin-induced amyloidosis distant from an injection site is unknown. Due to its rare nature, only a few case reports have been reported, with even fewer describing amyloidoma as distant from the insulin injection site. We present a case of a 52-year-old male with a left arm mass that was determined to be cutaneous amyloidosis and successfully treated with total excision of the mass. Histopathological examination with Congo red stain demonstrated classic characteristics of amyloidosis. We present this case report to increase awareness of this relatively rare occurrence.

## Introduction

There are approximately 50 human diseases that involve amyloid protein misfolding. Amyloidosis can present systemically or locally at any age. In systemic amyloidosis, the deposits are derived from serum plasma proteins, while those found in localized amyloidosis arise from proteins produced by cells at the deposition site [1]. This helps demonstrate that diseases can be related to single-organ impairment or systemic illness. Amyloid is a pathological, complex assembly of abnormal fibrillary proteins. These abnormal proteins accumulate and aggregate together as insoluble plaques that result in tissue impairment and organ dysfunction that cause disease. Research using X-ray, electron diffraction, and cry-electron microscopy has described the folding pattern most often in beta-pleated sheet structures [1].

Amyloidoma represents a solitary, usually singular collection of amyloid protein that arises in patients with or without evidence of systemic amyloidosis [2]. Due to its mass-like presentation, it is also called tumoral amyloidosis. This localized deposition of amyloid can be light chain amyloidosis (AL-type) or amyloid serum A protein (AA type). Amyloidoma without systemic amyloidosis is considered the rarest form of tissue amyloid deposition [2]. Due to its rare nature, the incidence is not well-known. There is some research suggesting that this may represent an early warning for some patients as up to 7% of amyloidomas develop systemic amyloidosis [2].

Patients with type 2 diabetes mellitus are prone to develop amylin islet amyloid polypeptide (AIAPP amyloid) plaques in the pancreas due to its abnormally elevated production in pancreatic beta cells [3,4]. Diabetic patients may also present with extrapancreatic amyloid deposition, usually at the sites of repeated subcutaneous insulin injections [4-7]. These amyloid deposits, primarily composed of the 37-residue islet amyloid polypeptide (IAPP), are a characteristic feature found in more than 90% of patients with type 2 diabetes [3,4]. The islet amyloid derived from IAPP is associated with approximately 60% beta-cell deficit, increased beta-cell apoptosis, and islet failure in type 2 diabetes [3,4]. Diabetic patients may also exhibit extrapancreatic amyloid deposition, often at sites of repeated subcutaneous insulin injections [3]. It is extremely rare for extrapancreatic amyloid deposition in the tissues distant to insulin injection sites.

We present a rare case of insulin-induced amyloidosis or amyloidoma that slowly developed at a satellite location distant from the insulin pump site that required surgical excision in a patient with type 1 diabetes. We present this case to increase awareness of this rare occurrence.

## Case presentation

**Figure 1:**
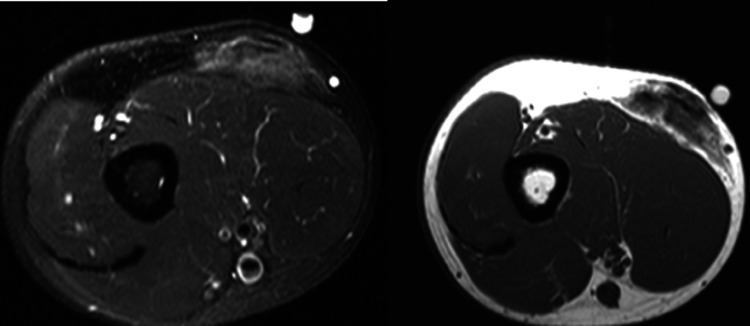
MRI of the upper extremity with and without contrast This image demonstrates a deep subcutaneous plane soft tissue mass located on the fascia lateral biceps mid-forearm. It shows faint T2 heterogeneous hyperintensity, with small foci of decreased signal possibly fibrosis, and less likely calcification. There is no invasion of deep fascia or muscle. Faint postcontrast enhancement was observed. Dimensions are approximately 1.6 x 3.8 x 4.6 cm (anteroposterior (AP) x transverse (TR) x craniocaudal (CC).

A 52-year-old male with a history of controlled type 1 diabetes mellitus since age 7 presented to the clinic with a firm, mobile, nodular mass on the distal third of his upper left arm that slowly developed over the last 10 years. He had been on an insulin pump until he switched to an Omnipod® in the past year to prevent a potential tubing obstruction while at work due to the physical demands of his job. His HgA1c has been less than 7.0 mg/dl for the past three years and has been well controlled. He reported that the lesion grew significantly larger and firmer in the last two years. He had no change in his insulin requirements and was not using this extremity for injections. He denied any pain or recent trauma to the area. An MRI of the left upper extremity was obtained, which showed a 4.6 cm maximum diameter mass lying on the fascia of the lateral biceps with decreased signal, suggesting possible fibrosis or calcification (Figure 1).

**Figure 2:**
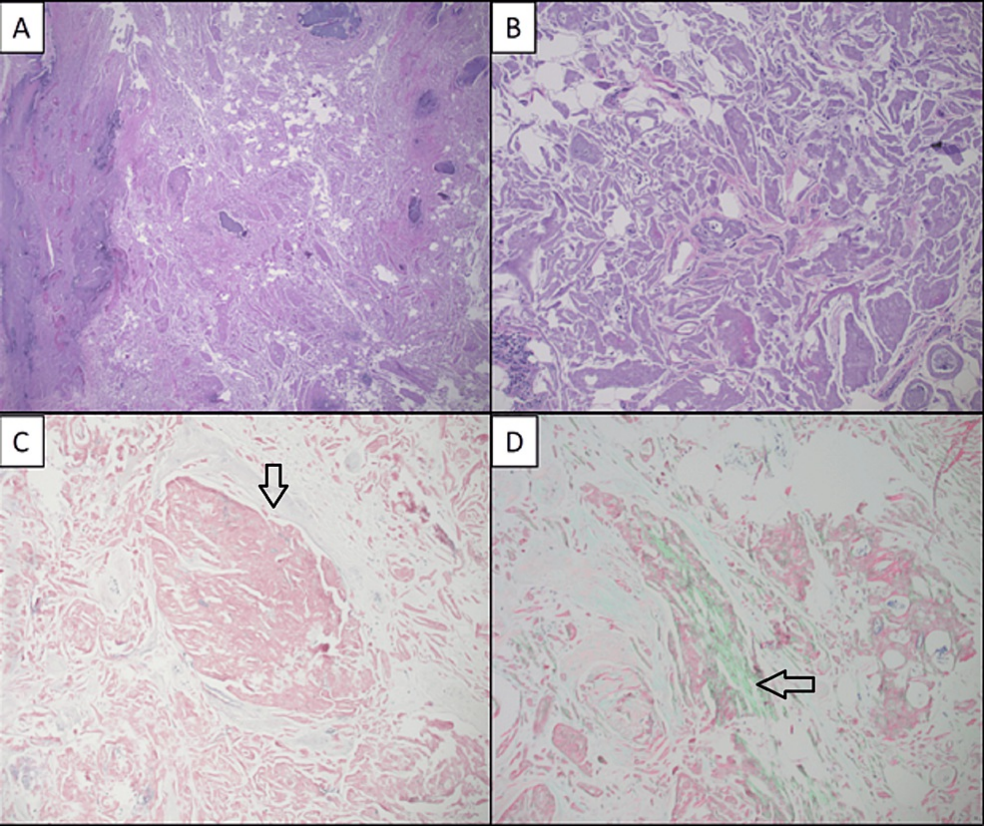
Histopathology of the left arm mass (A and B) H&E-stained sections show the aggregates of amorphous material with patchy mineralization (A: X20, B: X100). (C) Congo red stain highlighting the aggregates of amorphous material (X200). (D) Congo red stain under polarized light highlighting apple green birefringent amyloid (X200). H&E: Hematoxylin and eosin.

The lesion appeared to be confined to the subcutaneous tissue without involvement of the deep fascia or muscular tissue. A needle biopsy was attempted but was non-diagnostic. He was evaluated by a surgeon and underwent excision. During surgery, the lesion was found to be adherent to the fascia of the lateral biceps muscle, but the hard, calcified mass was successfully excised. Histologic sections of the mass show abundant aggregates of amorphous eosinophilic material. A Congo red stain was performed on paraffin sections of the specimen, which revealed Congo red-positive amyloid deposits, supporting amyloid deposition (Figure 2). Amyloid subtyping by liquid chromatography tandem mass spectrometry was subsequently performed confirming the AIns (insulin) subtype.

He had no evidence of systemic amyloidosis. After the surgery, he remained compliant with his medication regimen as prescribed and denied any recurrence of similar masses anywhere else on his body over a two-year time frame.

## Discussion

The first case of localized, AIns-type amyloidosis confined to the injection site was reported in 1988 by Endo et al. [5]. Since then, there have been more than 75 similar cases in patients using a wide variety of insulin formulations, suggesting that the incidence of insulin-derived amyloidosis is increasing [6]. The onset of insulin-derived amyloidosis ranges from several years to decades, with development affected by factors such as insulin dosing, injection technique, absorption, and clearance.

Given the growing prevalence of insulin-dependent diabetes, this number may actually be underreported [7]. There is a clinical overlap between insulin-derived amyloidosis and lipohypertrophy, which may lead to misdiagnosis [8]. Both are a result of repeated insulin injections; however, insulin-derived amyloidosis typically presents as a firmer mass that does not regress [9]. These masses tend to be solitary and irregularly shaped with ill-defined borders at the site of injections. The patient presented in our case vignette represents non-systemic, AIns-type amyloidosis located at a site distant from the abdominal insulin pump access port. The biopsy sample resulted in amyloid protein identification by mass spectrometry showing amyloidosis, insulin-type (AIns), consistent with iatrogenic localized amyloidosis associated with insulin injection areas in diabetic patients [9]. The patient has had regular follow-ups with excellent HgA1c control and no evidence of recurrence or systemic amyloidosis. It remains unclear if patients using continuous glucose monitors will have an increased risk of developing distal site amyloidoma.

The mechanism of amyloid formation at a satellite location is not clarified or well-known. Theories include the possibility of amyloid fibrils migrating through the bloodstream to distant sites and promoting amyloid aggregation by the process of seeding [10]. There appears to be an association of amyloid formation with poor diabetic control and an increase in insulin dose to achieve adequate blood sugar levels [11]. In a case series by Nagase et al., patients had a decrease in daily insulin requirements and lower blood glucose after changing their injection sites [12]. Serum insulin levels were found to be lower at amyloidosis sites [12]. Insulin fibrillation is largely a result of hydrophobic interactions among the molecules [13]. The misfolding of insulin proteins can lead to the aggregation of insoluble amyloid fibrils, which traps insulin and prevents absorption physically. Additionally, misfolded insulin has lower physiological activity that contributes to poor glycemic control. Injected insulin has been found to adhere strongly to amyloid fibrils, eventually undergoing transformation into amyloid as well [14].

With the expanding volume of literature on the topic, insulin-derived amyloidosis should now be considered a differential diagnosis for masses of unknown etiology in diabetic patients. To avoid the formation of amyloid in patients with insulin-dependent diabetes, medical practitioners should educate patients to routinely change the site of insulin injections. This may also be advantageous to ensure consistent subcutaneous absorption and allow for improved glycemic control. The rare incidence of both localized and distant amyloidosis formation warrants further exploration of its mechanism and other measures for prevention.

## Conclusions

We present a rare case of insulin-induced amyloidosis or amyloidoma, which slowly developed at a satellite location distant from the insulin pump site and required surgical excision in a patient with type 1 diabetes. We present this case to highlight this very rare occurrence and increase physician awareness.

Patients with diabetes are at risk of developing amyloidomas within the pancreas and at the insulin pump site or very rarely at a distant satellite location. Due to its rare nature, more information is needed to determine the actual risk of developing systemic amyloid, determine the risk of recurrence of the lesion, and establish guidelines for surveillance.
